# Association Between Systemic Symptoms and Recovery in Acute Low Back Pain: A Retrospective Cross-Sectional Study

**DOI:** 10.3390/jcm14196969

**Published:** 2025-10-01

**Authors:** Ji-Ho Lee, Si-Hyun Han, Min-Su Kim, Dong-Ho Keum, Seo-Hyun Park

**Affiliations:** 1 College of Korean Medicine, Dongguk University Graduate School, Seoul 04620, Republic of Korea; wigh1996@naver.com; 2 Department of Rehabilitation Medicine of Korean Medicine, Dongguk University Bundang Oriental Hospital, Seongnam-si 13601, Republic of Korea; h4692@naver.com (S.-H.H.); alstn7180@naver.com (M.-S.K.); keumdh660@naver.com (D.-H.K.); 3 College of Korean Medicine, Dongguk University Graduate School, Gyeongju 38066, Republic of Korea

**Keywords:** low back pain, prognosis, chronicity, systemic symptom, dyspepsia

## Abstract

**Background**: Several prognostic factors, including the early recovery pattern of acute low back pain (ALBP), are related to the chronicity of LBP. However, the association between systemic symptoms and ALBP remains underexplored from a holistic perspective. Hence, this study aimed to investigate this relationship and identify novel clinical prognostic predictors for LBP. **Methods**: This retrospective cross-sectional study included patients with ALBP admitted to the Department of Korean Medicine Rehabilitation at the Dongguk University Bundang Hospital between 1 January 2021 and 30 April 2025. Data extracted from medical records included demographics, treatment-related information, pain characteristics, past medical history, and systemic symptoms. Statistical analyses included independent *t*-tests, Mann–Whitney U tests, chi-squared tests, Fisher’s exact tests, correlation analysis, and multiple linear regression models. **Results**: A total of 194 patients with ALBP were included in the analysis. Among systemic symptoms, dyspepsia was significantly associated with higher pain at discharge and smaller absolute and relative pain changes. Although sleep disturbance and constipation showed associations with higher pain at discharge in univariate analyses, these associations were not statistically significant in regression models. Beyond systemic symptoms, alcohol consumption was significantly associated with lower pain at discharge and greater relative pain change, while hospitalization and symptom duration exhibited non-linear relationships. These findings remained robust in sensitivity and subgroup analyses. **Conclusions**: Systemic symptoms, especially dyspepsia, may serve as prognostic factors impeding ALBP recovery, representing potential early markers for identifying patients at risk of chronicity. The findings highlight the prospect of multidimensional strategies in reducing pain and enhancing patients’ quality of life in clinical practice.

## 1. Introduction

Low back pain (LBP) is defined as pain or discomfort localized below the costal margin and above the inferior gluteal folds, which can radiate to the lower extremities. LBP is a commonly encountered musculoskeletal symptom in primary care, with a reported lifetime prevalence of 49–70% [1]. Among various LBP classification systems, the etiological classification distinguishes mechanical from non-mechanical causes. Mechanical LBP, accounting for approximately 97% of cases, arises from structural or functional abnormalities of the spine and surrounding tissues, whereas non-mechanical LBP, comprising the remaining 3% of cases, is caused by extrinsic pathologies such as infections, malignancies, or visceral disorders [2]. LBP can also be classified according to symptom duration. Although somewhat controversial, acute LBP is generally defined as pain lasting <4–6 weeks, while subacute and chronic phases are subsequently delineated using a 12-week threshold [1,3]. Over 90% of patients with acute LBP (ALBP) recover fully within days to weeks, whereas approximately 5% progress to chronic LBP (CLBP) via the subacute phase [4].

CLBP impairs not only the patient’s physical health but also psychological well-being and socioeconomic functioning. Studies showed that CLBP is frequently accompanied by sleep disturbances, depression, and anxiety [5,6,7]. Additionally, productivity loss imposes a substantial societal LBP-related burden [8]. Moreover, despite the availability of various treatment modalities reflecting the multifactorial nature of CLBP, treatment efficacy remains inconsistent, and the factors influencing the therapeutic response are not yet fully understood [9]. Consequently, a small subset of patients with CLBP accounts for nearly 80% of the total healthcare costs for LBP [9].

Given the considerable socioeconomic burden imposed by CLBP, early identification of patients at risk for chronicity is of utmost clinical importance. Studies demonstrated that CLBP is more strongly related to demographic, psychosocial, and occupational factors than with musculoskeletal pathology alone [10]. These findings prompted a shift toward the biopsychosocial model, which integrates biological, psychological, and social dimensions in the understanding of pain perception [11]. Several prognostic factors for chronicity, including sex, age, obesity, physical workload and awkward posture, social isolation, depression, diabetes, and smoking, have been proposed [12].

Furthermore, the short-term progression of ALBP is recognized as an indicator of chronicity risk. Recovery patterns within the first week of onset predict the LBP course at 1 and 3 months with significant accuracy [13]. Moreover, most patients with LBP who continued to experience activity limitations for 3 months from the onset had already exhibited signs of delayed recovery within the first week [14]. Therefore, early recovery patterns may serve as valuable prognostic indicators of long-term outcomes in ALBP.

Beyond the biopsychosocial model, a holistic approach has recently gained attention, seeking to investigate the relationship between LBP and systemic symptoms. Systemic symptoms, identified through comprehensive history-taking, refer to symptoms arising from other organ systems unrelated to the patient’s chief complaint [15]. These symptoms are utilized to assess the overall health status, aid in differential diagnosis, and evaluate the systemic impact of diseases [15]. Studies reported a higher risk of LBP in women with frequent gastrointestinal (GI) symptoms, as well as increased rates of urinary incontinence or GI symptoms among women with LBP [16,17]. Furthermore, women with LBP who experience constipation exhibit higher pain sensitivity [18]. However, these studies did not examine the impact of systemic symptoms on the clinical LBP course. Sleep disturbances also demonstrated a bidirectional association between pain intensity, which may contribute to delayed recovery [19].

Traditional Korean Medicine (TKM) adopts a holistic diagnostic and therapeutic approach, encompassing physiological characteristics, lifestyle factors, and systemic symptoms. The *Donguibogam*, a foundational text in TKM, reflects a longstanding recognition of the association between LBP and systemic symptoms, classifying LBP into subtypes based on accompanying symptoms: urinary dysfunction (kidney deficiency type), GI symptoms (food accumulation type), and psychological symptoms (Qi stagnation type) [20]. Accordingly, TKM practitioners comprehensively assess systemic symptoms (i.e., sleep patterns, dietary habits, digestive function, bowel and urinary symptoms, and thirst) in conjunction with pain intensity, duration, and location [20]. This multidimensional information determines the underlying pathophysiology of pain and supports pattern identification and treatment planning. From this holistic perspective, systemic symptoms may plausibly influence LBP prognosis. Indeed, the prognostic value of systemic symptoms for neck pain was reported [21]. However, no studies have examined the prognostic value of systemic symptoms in ALBP.

This study retrospectively analyzed systemic symptoms in inpatients with ALBP, aiming to identify potential novel factors that may significantly influence early recovery patterns. Specifically, we aimed to evaluate the prognostic value of systemic symptoms commonly used in TKM and explore the clinical relevance of a holistic approach through univariate and multivariate statistical analyses. Given that short-term progression can predict long-term outcomes of LBP, this study is expected to provide early indicators for identifying the risk of chronicity.

## 2. Materials and Methods

### 2.1. Study Design and Eligibility Criteria

This retrospective cross-sectional study aimed to investigate potential factors influencing recovery from ALBP by analyzing the correlation between ALBP and systemic symptoms. The Institutional Review Board (IRB) of Dongguk University Bundang Oriental Hospital approved this study (IRB no. 2025-0005). All procedures adhered to the Strengthening the Reporting of Observational Studies in Epidemiology (STROBE) guidelines [22]. Patients who were admitted to the Department of Korean Rehabilitation Medicine for acute mechanical LBP between 1 January 2021 and 30 April 2025, were included in the study. LBP diagnoses were identified using the Korean Standard Classification of Diseases (KCD) diagnostic codes recorded at initial admission, specifically S30–S39 (injury to the abdomen, lower back, lumbar spine, and pelvis) and M50–M54 (other dorsopathies).

The following eligibility criteria were applied to ensure data reliability and sample consistency (Table 1). First, patients with non-mechanical LBP (such as spinal tumor, spinal infection, or ankylosing spondylitis) were excluded to focus exclusively on mechanical LBP. Mechanical LBP is generally defined as pain arising from structural or functional abnormalities of the spine and its surrounding tissues. However, this study also classified cases involving radicular pain as mechanical LBP when nerve root compression could be clearly attributable to structural lesions, such as disc herniation or spinal stenosis [2].

Furthermore, to minimize potential confounding, patients aged > 65 years, presenting with red flag signs, or with concussion at LBP onset were excluded. Older patients are subject to various confounders (including age-related degenerative changes, comorbidities, and psychosocial issues such as anxiety or cognitive impairment), which may substantially influence the prognosis of LBP [23,24]. Red flag signs indicate potential serious underlying conditions such as compression fractures, cauda equina syndrome, progressive neurological deficits, infection, or malignancy [25]. Among these, compression fractures and cauda equina syndrome, although structural in nature, were excluded due to their distinct clinical course and specific management strategies (i.e., urgent surgical intervention in cauda equina syndrome), which differ significantly from those for typical mechanical LBP [26,27,28]. Lastly, concussion was excluded as it may alter pain perception through central sensitization, compromising the reliability of self-reported pain measures [29,30].

Moreover, patients who received no treatment during hospitalization or were admitted for ≤3 days were excluded due to insufficient therapeutic exposure to ensure homogeneity of therapeutic outcomes. Furthermore, although ALBP is typically defined as <4–6 weeks, a more conservative cutoff of 4 weeks was adopted to avoid the inclusion of cases possibly transitioning into the subacute phase by the final assessment [2].

### 2.2. Data Extraction

Patient’s medical records were reviewed to obtain comprehensive data. A third-party independent of the study extracted the data and removed personal identifiers to ensure patient anonymity and data protection. The processed data were subsequently provided to two independent researchers for verification and organization. Any disagreements between the researchers were resolved through discussion. Then, the finalized data set was transferred to another researcher for statistical analysis.

Demographic data were retrieved from the admission records, including sex, age, symptom duration, hospitalization length, diagnosis, smoking and alcohol history, and past medical history. The latter encompassed a history of hypertension (HTN), dyslipidemia, diabetes mellitus (DM), cardiovascular disease, gynecological disease, upper and lower GI disease, respiratory disease, musculoskeletal diseases (thoracolumbar, cervical, or other regions), and psychological history (PSY). The classification of upper and lower GI disorders was based on the anatomical landmark known as the ligament of Treitz, with disorders from the esophagus to the duodenum categorized as upper GI, whereas those from the jejunum to the anus were categorized as lower GI [31]. Only conditions with confirmed diagnoses in the hospital were included.

Details about LBP were collected from admission and discharge records, including pain intensity, pain difference, pain distribution, and range of motion (ROM). Pain intensity was examined using the Numeric Rating Scale (NRS) both at admission (NRS AD) and discharge (NRS DC). Pain difference was calculated as both an absolute change (△NRS=NRS DC−NRS AD) and a relative change (△NRS[%]=(NRS DC−NRS AD)/NRS AD×100).

Systemic symptoms were identified from the admission and progress records as patient-reported symptoms based on patient interviews conducted by Korean medicine doctors with at least one year of clinical experience. This study focused on six systemic symptoms listed in the hospital records: sleep disturbance, anorexia, dyspepsia, constipation, nocturia, and thirst. Given their subjective nature, relevant diagnostic criteria and literature were referenced, if applicable, to enhance the objectivity and clinical relevance of their interpretation [32,33,34,35]. Table 2 presents the detailed definitions.

### 2.3. Statistical Analysis

All statistical analyses were primarily conducted using IBM SPSS Statistics for Windows, Version 21 (IBM Corp., Armonk, NY, USA). Regression analyses were performed using R Studio Version 4.2.0. Continuous variables were presented as means and standard deviations, while categorical data were presented as frequencies and percentages. Binary variables included sex, smoking history, alcohol consumption, presence of past medical history, pain distribution, and presence of each systemic symptom. ROM was also classified as a binary variable (normal and abnormal) based on the hospital medical record system, with ‘normal’ defined as no limitation in any lumbar ROM.

The dependent variable was the pain score measured by the NRS, a continuous variable ranging from 0 to 10, with higher scores indicating greater pain severity [36]. Four subscales were analyzed: NRS AD, NRS DC, absolute pain change (ΔNRS), and relative pain change (ΔNRS [%]). Improvement was indicated by a lower NRS DC compared to NRS AD. Accordingly, negative values in both absolute and relative changes reflected pain relief, while positive values indicated worsening of pain.

Univariate analyses were conducted using the independent *t*-test or Mann–Whitney U test, depending on the normality of each independent variable. Normality was assessed through the Kolmogorov–Smirnov or Shapiro–Wilk test, depending on the sample size. Spearman or Pearson correlation coefficients were computed to examine the correlations between continuous variables, as appropriate. Chi-square or Fisher’s exact tests were employed to evaluate the associations between binary variables. A *p*-value < 0.05 with a 95% confidence interval indicated statistical significance.

Multiple linear regression analyses were conducted to identify the independent effects of each variable on the LBP progression. Variables with statistical significance (*p* < 0.05) or suggestive trends (*p* < 0.1) in univariate analyses were included in the model. Model assumptions of linearity, normality of residuals, homoscedasticity, and multicollinearity were assessed to ensure validity. Generalized additive models (GAMs) were applied to account for potential non-linear relationships for continuous variables violating the linearity assumption. Subsequently, bootstrapping was employed to estimate confidence intervals for the regression coefficients for any remaining violations of normality and homoscedasticity. Further sensitivity and subgroup analyses were conducted to assess the robustness of the findings. Sensitivity analyses used two alternative models: (1) one including all variables and (2) one incorporating only known predictors of LBP chronicity. The same procedures were followed without bootstrapping, focusing on consistency in coefficient direction and significance. Subgroup analyses were conducted based on patients’ diagnoses.

## 3. Results

### 3.1. General Characteristics of Participants

**Figure 1:**
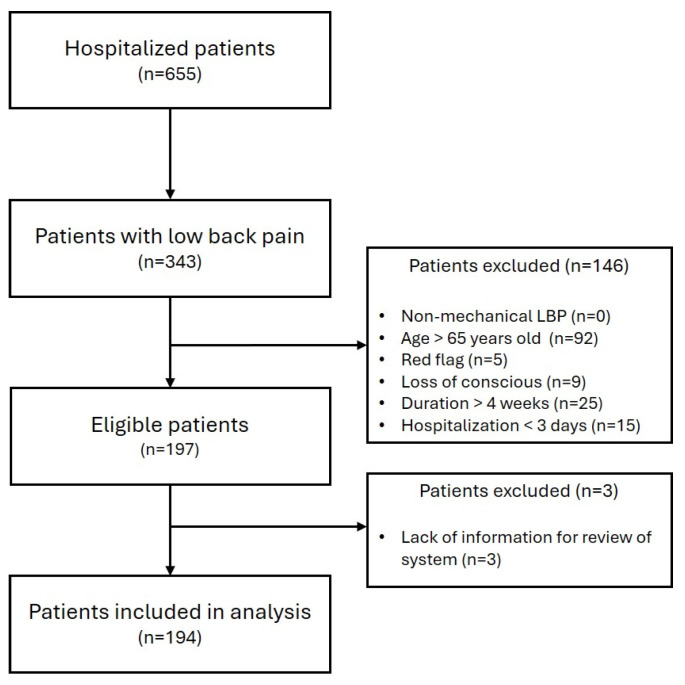
Flow chart of patient selection. Abbreviation: LBP, Low back pain.

A total of 655 patients were admitted to the clinic between 1 January 2021 and 30 April 2025. Among them, 343 patients met the study criteria for LBP, whereas 194 eligible patients with ALBP were included in the statistical analysis (Figure 1). Within this cohort, 79 patients were male, and 115 patients were female, with a mean age of 47.06 ± 12.11 years. The average complaint duration was 2.65 ± 4.18 days, and the mean hospitalization length was 6.57 ± 3.84 days. Regarding diagnoses, the majority of patients were classified under “sprain and strain of lumbar spine” (*n* = 181), followed by “other and unspecified sprains and strains of lumbar and pelvic region” (*n* = 8) and “lumbar and other intervertebral disc disorders with radiculopathy” (*n* = 5). The mean NRS AD was 6.09 ± 1.46, and the mean NRS DC was 4.77 ± 2.01. The average absolute pain change (ΔNRS) was –1.32 ± 1.75, and the average relative change (ΔNRS [%]) was –21.61 ± 27.93% (Table 3).

### 3.2. Association Between Demographic Characteristics and Pain Intensity

Given the violation of normality, the Mann–Whitney U test and Spearman’s rank correlation were applied to binary variables (gender, smoking, and alcohol) and continuous variables (age), respectively. Among the four variables, only smoking and alcohol consumption demonstrated a significant association with pain intensity at discharge and pain reduction (*p* < 0.05 or *p* < 0.01). None of the variables showed significant associations with initial pain intensity (Table 4A).

### 3.3. Association Between Past Medical History and Pain Intensity

Given the violation of normality, the Mann–Whitney U test was conducted. Patients with a history of thoracolumbar musculoskeletal disorders exhibited significantly higher initial pain intensity (*p* < 0.05). Additionally, those with a history of lower GI disorders showed a suggestive trend toward lower initial pain (*p* < 0.1). However, no past medical history was significantly related to pain at discharge or overall pain change (Table S1)

### 3.4. Association Between Low Back Pain Characteristics and Pain Intensity

Given the violation of normality, the Mann–Whitney U test and Spearman’s rank correlation were applied according to variable types. Initial pain intensity (NRS AD) was positively correlated with pain at discharge (*p* < 0.001) and inversely correlated with absolute pain change (*p* < 0.05) but was not associated with relative pain change. Symptom duration and hospitalization length showed a negative correlation with pain at discharge and both absolute and relative pain change (symptom duration, *p* < 0.001; hospitalization length, *p* < 0.05 or *p* < 0.01). Pain distribution did not significantly influence pain at discharge or pain change, although cases of widespread pain demonstrated a suggestive trend toward higher initial pain (*p* < 0.1). Finally, abnormal ROM was significantly associated only with initial pain and showed a marginal trend toward the association with higher pain at discharge (*p* < 0.1) (Table 4B).

### 3.5. Association Between Systemic Symptoms and Pain Intensity

Given the violation of normality, the Mann–Whitney U test was conducted. Sleep disturbance was significantly associated with higher pain at discharge (*p* < 0.05) but showed no significant difference in initial pain or pain change. Constipation showed a trend toward higher pain at discharge (*p* = 0.076), while dyspepsia exhibited trends toward smaller absolute and relative pain changes (*p* = 0.052 and 0.054). However, neither significantly influenced initial pain. Other systemic symptoms showed no significant association with pain intensity or pain change (Table 4C).

### 3.6. Association Between Systemic Symptoms and Pain Characteristics

Chi-square tests were conducted to examine the associations between systemic symptoms and LBP characteristics. Constipation and dyspepsia showed a suggestive association with widespread pain distribution (*p* < 0.1). No other significant associations were identified (Table S2).

### 3.7. Multiple Regression Analysis

The independent variables of the basic regression model included hospitalization length, symptom duration, initial pain intensity (NRS AD), sleep disturbance, dyspepsia, constipation, history of lower GI disorders, history of thoracolumbar musculoskeletal disorders, pain distribution, smoking status, alcohol consumption, and ROM. The dependent variables were pain at discharge (NRS DC), absolute pain change (ΔNRS), and relative pain change (ΔNRS [%]). Table 5 presents the results of multiple regression.

#### 3.7.1. Pain at Discharge

Violations of the linearity, residual normality, and homoscedasticity assumptions were identified through the residuals vs. fitted plot (Figure S1a), the Shapiro–Wilk normality test (*p* = 0.003), and the Breusch-Pagan test (*p* = 0.026). However, all variance inflation factors (VIFs) were <2, indicating no multicollinearity. Partial residual plots of continuous variables revealed non-linearity in hospitalization and symptom duration, for which GAMs were subsequently applied for correction (Figure S2a). Upon re-evaluation, residual diagnostics continued to show normality and heteroscedasticity violations, as indicated by the Shapiro–Wilk test (*p* = 0.004) and the residuals vs. fitted plot. Accordingly, bootstrapping was employed to obtain robust confidence intervals.

**Figure 2:**
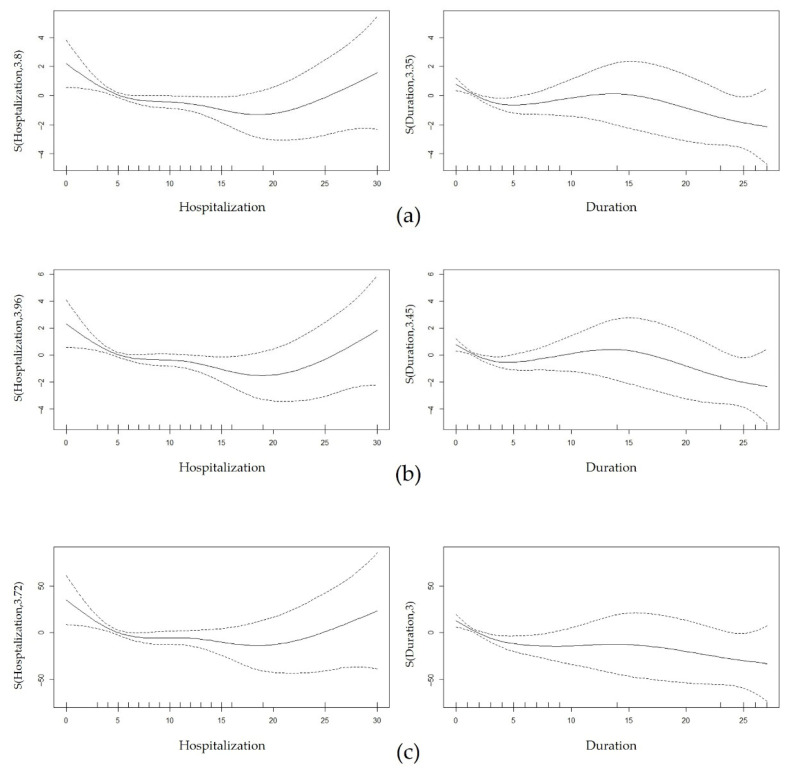
Graphs of estimated smooth effect of hospitalization (**left**) and duration (**right**) using GAM. (**a**) NRS DC, (**b**) ΔNRS, (**c**) ΔNRS (%). The solid line indicates the estimated smooth function, and the dashed lines represent the 95% confidence interval. The *x*-axis represents the number of days since hospitalization or symptom onset. The *y*-axis depicts the estimated smooth function, representing the non-linear effect of the predictor on the outcome while adjusting for other covariates. Deviations from zero indicate the direction and strength of this effect. The curve suggests a non-linear association between the two variables. Abbreviation: GAM, Generalized Additive Model; NRS, Numeric rating scale; NRS DC, NRS at discharge; ΔNRS, absolute pain change = NRS DC − NRS AD; ΔNRS (%), relative pain change = ((NRS DC − NRS AD)/NRS AD) × 100.

The model included 194 patients, with an adjusted R^2^ of 0.417 and deviance explained at 46.9%. Initial pain intensity (*β* = 0.71, 95% CI: 0.56–0.90, *p* < 0.001) and dyspepsia (*β* = 0.55, 95% CI: 0.05–1.02, *p* < 0.05) were significantly associated with higher pain at discharge. In contrast, alcohol consumption was significantly associated with lower pain at discharge (*β* = −0.58, 95% CI: −1.06–−0.03, *p* < 0.05). Furthermore, hospitalization length (EDF = 3.80) and symptom duration (EDF = 3.35) exhibited significant non-linear effects (Figure 2a). No other variables showed significant associations.

#### 3.7.2. Absolute Pain Change

NRS AD was excluded from the model as an independent variable since the absolute change was defined as NRS DC − NRS AD, which would induce mathematical coupling. Violations of the linearity and residual normality assumptions were identified through the residuals vs. fitted plot (Figure S1b) and the Shapiro–Wilk normality test (*p* = 0.000). The Breusch-Pagan test supported homoscedasticity (*p* = 0.102); however, the residuals versus fitted values plot suggested a potential violation. VIFs were all <2, indicating no evidence of multicollinearity. Non-linearity in hospitalization and symptom duration was identified via partial residual plots, and GAMs were applied for correction (Figure S2b). Bootstrapping was employed to derive robust confidence intervals since the Shapiro–Wilk test (*p* = 0.003) and the residuals vs. fitted plot indicated persistent normality and heteroscedasticity violation.

The model included 194 patients, with an adjusted R^2^ of 0.176 and deviance explained at 24.6%. Dyspepsia was significantly associated with smaller absolute pain change (*β* = 0.54, 95% CI: 0.06–1.00, *p* < 0.05). In contrast, alcohol consumption (*β* = −0.50, 95% CI: −1.01–0.05, *p* < 0.1) and smoking (*β* = −0.64, 95% CI: −1.54–0.13, *p* < 0.1) showed suggestive associations with greater pain change, although their bootstrapped confidence intervals included zero, indicating uncertainty in these estimates. Additionally, hospitalization length (EDF = 3.96) and symptom duration (EDF = 3.45) exhibited significant non-linear effects significant non-linear effects (Figure 2b). No other variables showed significant associations.

#### 3.7.3. Relative Pain Change

Given that relative change was defined as (NRS DC − NRS AD)/NRS AD, NRS AD was excluded from the model as an independent variable to avoid endogeneity and potential bias in the regression estimates. Violations of the linearity and residual normality assumptions were identified by the residuals vs. fitted plot (Figure S1c) and the Shapiro–Wilk normality test (*p* = 0.003). Although the Breusch-Pagan test showed no significant heteroscedasticity (*p* = 0.2825), the residual plot suggested a potential mild dichotomous distribution. VIFs for all variables were <2, indicating no multicollinearity concerns. Partial residual plots revealed non-linearity of hospitalization and symptom duration, which was addressed using GAMs (Figure S2c). Bootstrapping was employed to adjust confidence intervals since the Shapiro–Wilk test indicated a persistent violation of normality (*p* = 0.008).

The model included 194 patients, with an adjusted R^2^ of 0.168 and deviance explained at 23.6%. Dyspepsia showed a significant association with smaller relative pain change (*β* = 8.15, 95% CI: 0.27–16.38, *p* < 0.05). In contrast, alcohol consumption was significantly associated with a greater pain change (*β* = −11.57, 95% CI: −19.33–−2.97, *p* < 0.05). Furthermore, hospitalization length (EDF = 3.72) and symptom duration (EDF = 3.00) exhibited significant non-linear effects (Figure 2c). No other variables showed significant associations.

#### 3.7.4. Sensitive and Subgroup Analysis

Table S3 presents the results of the sensitivity and subgroup analysis of multiple regression. In the full model including all covariates, the regression coefficient for dyspepsia remained consistent in both direction and magnitude across all pain outcomes (NRS DC: *β* = 0.66, *p* < 0.05; ΔNRS: *β* = 0.67, *p* < 0.05; ΔNRS [%]: *β* = 10.21, *p* < 0.05). Alcohol consumption also showed a consistent association with pain at discharge and relative pain change (NRS DC: *β* = −0.66, *p* < 0.05; ΔNRS [%]: *β* = −13.13, *p* < 0.01), while absolute pain change demonstrated only a suggestive association (ΔNRS: *β* = −0.57, *p* < 0.1). Initial pain intensity retained a significant association with pain at discharge (NRS DC: *β* = 0.71, *p* < 0.001). Additionally, suggestive associations were observed for smoking (NRS DC: *β* = −0.72, *p* < 0.1; ΔNRS: *β* = −0.75, *p* < 0.1), age (NRS DC: *β* = −0.02, *p* < 0.1, ΔNRS [%]: *β* = −0.45, *p* < 0.1), and ROM (ΔNRS [%]: *β* = 6.62, *p* < 0.1).

The regression coefficient for dyspepsia (NRS DC: *β* = 0.69, *p* < 0.01; ΔNRS: *β* = 0.69, *p* < 0.01; ΔNRS [%]: *β* = 10.43, *p* < 0.05) and alcohol consumption (NRS DC: *β* = −0.70, *p* < 0.05; ΔNRS: *β* = −0.63, *p* < 0.05; ΔNRS [%]: *β* = −13.64, *p* < 0.01) remained consistent in both direction and magnitude across all pain outcomes in the model adding known predictors of LBP chronicity (age, gender, psychological history, DM, and HTN). Initial pain intensity remained significant for pain at discharge (NRS DC: *β* = 0.71, *p* < 0.001). Additionally, smoking showed significant or suggestive associations (NRS DC: *β* = −0.75, *p* < 0.05; ΔNRS: *β* = −0.77, *p* < 0.1).

Subgroup analysis was performed with the basic model, including 181 patients diagnosed with lumbar sprain or strain. The regression coefficient for dyspepsia was consistently maintained across all pain outcomes, although the association reached only suggestive significance (NRS DC: *β* = 0.49, *p* < 0.1; ΔNRS: *β* = 0.48, *p* < 0.1; ΔNRS [%]: *β* = 7.23, *p* < 0.1). Initial pain intensity remained strongly associated with pain at discharge (NRS DC: *β* = 0.71, *p* < 0.001), whereas alcohol consumption showed a significant or suggestive association with pain at discharge and relative pain change (NRS DC: *β* = −0.49, *p* < 0.1; ΔNRS [%]: *β* = −10.70, *p* < 0.05).

#### 3.7.5. Association Between Binary Variables Included in Regression

Although the absence of multicollinearity was observed in the regression analysis, a Chi-squared test was additionally conducted to examine the independence between categorical variables. Alcohol consumption and smoking demonstrated a strong association (*p* < 0.001). Furthermore, significant associations were observed between dyspepsia and constipation, dyspepsia and widespread pain, past history of lower GI disorders and thoracolumbar musculoskeletal disorders, and past history of thoracolumbar musculoskeletal disorders and widespread pain (*p* < 0.05) (Table S4).

## 4. Discussion

This retrospective study analyzed 194 patients hospitalized for ALBP to investigate how systemic symptoms influence the clinical course of ALBP. Progression to CLBP causes functional impairment and psychosocial issues such as sleep disturbance, anxiety, and depression while imposing a long-term socioeconomic burden through reduced productivity and increased healthcare costs [5,6,7,8,9]. Accordingly, numerous studies explored prognostic factors for the early prevention of LBP chronicization, emphasizing psychosocial and demographic variables, as well as the short-term recovery of ALBP [13,14]. However, to the best of our knowledge, no studies have examined systemic symptoms as prognostic factors for LBP through a holistic perspective. Recognizing that short-term recovery of ALBP may predict its chronicization, this study assessed the impact of systemic symptoms on the clinical course of ALBP, ultimately identifying long-term prognostic factors and highlighting the value of a holistic approach.

Among the systemic symptoms, dyspepsia may hinder recovery from ALBP as an independent risk factor. In univariate analysis, dyspepsia showed a suggestive association with higher pain at discharge (*p* = 0.091) and lower absolute (*p* = 0.052) and relative (*p* = 0.054) change in pain. However, dyspepsia was significantly associated with greater pain at discharge (*β* = 0.55, *p* = 0.026, 95% CI [0.05, 1.02]) and lower pain improvement both in absolute (*β* = 0.54, *p* = 0.033, 95% CI [0.06, 1.00]) and relative terms (*β* = 8.15, *p* = 0.045, 95% CI [0.27, 16.38]) in regression analysis. The observed discrepancy likely reflects the limitations of univariate analysis in accounting for confounding and interaction effects, whereas regression models adjust for these influences, providing a more precise estimate of the independent effect.

Dyspepsia also demonstrated a robust association in the sensitivity analyses, demonstrating consistent effects regardless of variable selection or analytic conditions. Dyspepsia consistently showed significant associations across all pain outcomes in the full model and the model with known chronicity factors, highlighting its robustness and novelty as an independent factor beyond previously identified predictors. Furthermore, dyspepsia exhibited suggestive associations across pain outcomes in subgroup analysis. Nevertheless, the directions of the coefficient remained consistent with that of the base model. Additionally, the regression coefficients for absolute pain change associated with dyspepsia were estimated at 0.48–0.69, indicating a corresponding reduction in the extent of LBP improvement. Given the average admission pain score of 6.09 ± 1.46 and the reported Minimal Clinically Important Difference (MCID) of −2 for moderate LBP (NRS 5–7), dyspepsia may meaningfully hinder the degree of pain reduction required to meet the MCID threshold [37]. Hence, the adjusted R^2^, representing the explanatory power of the base regression model, ranged from 16.8% to 41.7%. Consequently, these results suggest a reasonable level of reliability since R^2^ values >15% are generally considered meaningful in clinical research [38]. Taken together, these statistical and clinical interpretations suggest that dyspepsia may serve as a significant independent factor impeding the recovery of ALBP and potentially contributing to its chronicity.

No studies have yet reported an influence of dyspepsia on ALBP prognosis. Moreover, a definitive pathophysiological mechanism has yet to be established. Nevertheless, several plausible mechanisms can be proposed based on anatomical and physiological considerations. First, hyperactivation of the abdominal muscles, including the rectus abdominis, external oblique, and internal oblique, is frequently observed in LBP patients [39]. This increases intra-abdominal pressure (IAP) as a compensatory mechanism, enhancing spinal stability and reducing the mechanical load on the vertebrae [40,41]. The elevated IAP may concurrently reduce gastrointestinal blood flow, thereby contributing to dyspeptic symptoms [42]. Second, dyspepsia may impact LBP through viscerosomatic reflexes. The upper GI tract is primarily innervated by the T5–T9 spinal segments. Visceral stimuli from GI symptoms are conveyed via visceral afferent fibers to the corresponding spinal dorsal horn segments, where they may affect somatic nerves through viscerosomatic convergence [43]. Such convergence can trigger reflexive muscle tension in dorsal rami–innervated muscles at the same spinal segments [44]. These muscles, including the erector spinae, multifidus, and intertransversarii, play a critical role in segmental spinal stability and fine motor control [44]. As these muscles are anatomically integrated with the thoracolumbar fascia, sustained reflexive tension may induce fascial stress and deformation. Given previous evidence that thoracolumbar fascia deformation may contribute to the onset of ALBP, visceral stimulation associated with dyspepsia could exacerbate pain or hinder recovery [45]. Lastly, dyspepsia may induce central sensitization, leading to altered pain perception. In visceral pain disorders such as irritable bowel syndrome or functional dyspepsia, cortical reorganization has been reported in the anterior cingulate cortex (ACC) and medial prefrontal cortex [46]. These alternations affect sensory perception, pain modulation, and emotional as well as cognitive processing [46]. The ACC, in particular, is activated by acute visceral pain through glutamatergic signaling, contributing to the formation of pain memory and negative affective responses [47]. These neural changes induced by visceral pain can increase overall pain sensitivity, potentially amplifying the perception of pain arising from other regions [48]. Consequently, LBP patients with dyspepsia may experience more intense and persistent pain.

Constipation is another systemic symptom warranting attention. While univariate analysis indicated a suggestive association with higher pain at discharge (*p* = 0.076), no significant effects were observed in absolute or relative pain change across analyses. However, mean comparisons suggested a substantial negative effect of constipation on LBP recovery. Patients with constipation showed a 0.6-point smaller absolute reduction and a 12% lower relative improvement in pain compared to controls. These differences may be clinically meaningful because dyspepsia, a systemic symptom previously discussed with a suggestive association in univariate analysis, showed only a 0.5-point and 8% difference. The lack of statistical significance may reflect the limited power due to the small number of patients with constipation (8 of 194, 4.1%). Moreover, several studies reported a significant association between constipation and increased LBP intensity [18,49], attributing it to the increased mechanical load from the elevated intra-abdominal pressure during defecation and altered pain modulation due to gut microbiome dysbiosis [18,49]. Taken together, the present findings do not entirely exclude a potential association between constipation and ALBP prognosis. Thus, further studies with larger sample sizes are recommended to clarify the clinical impact of constipation on ALBP prognosis.

Regarding sleep disturbance, a significant association with higher pain at discharge was identified only in univariate analysis (*p* = 0.049), while no significant findings emerged for absolute or relative pain change in any analyses. This finding does not fully align with prior studies indicating a bidirectional correlation between sleep disturbance and LBP, highlighting that poor sleep quality elevates next-day pain, whereas higher pain intensifies sleep disturbance, creating a vicious cycle [19]. This discrepancy may be partly attributable to the measurement of sleep disturbance. Although identified from clinical records referencing diagnostic criteria, sleep disturbances were based on subjective complaints rather than standardized tools, such as sleep diaries and the Pittsburgh Sleep Quality Index, potentially limiting diagnostic accuracy and influencing the results. Nevertheless, given that sleep disturbance exhibited consistently positive, but not significant, regression coefficients across all pain outcomes, it may interact with other clinical factors in a more complex manner. However, this finding alone cannot conclusively establish the effect due to conflicting evidence on the impact of sleep disturbances on LBP [13,19]. Therefore, further research employing rigorous and standardized sleep assessment tools is needed.

Among demographic characteristics, both smoking and alcohol consumption were significantly associated with lower pain at discharge and greater pain change in univariate analysis. However, only alcohol remained significant in multivariate regression, suggesting greater explanatory relevance. Given the strong association between the two (chi-square, *p* = 0.000), the effect of smoking may reflect the influence of alcohol consumption due to shared variance, with its independent contribution attenuated in the regression model despite the absence of multicollinearity. The observed effect of alcohol aligns with prior findings showing that moderate consumption is associated with a reduced risk of pain chronicity, lower pain intensity and extent, and enhanced physical function, especially in non-localized pain conditions [50,51]. However, the apparent analgesic effect of alcohol may reflect elevated pain thresholds rather than actual improvement in mechanical pain. Indeed, consuming 3–4 drinks at least once per month is associated with significantly higher pain thresholds compared to non-drinkers [52]. Therefore, the potential benefits of alcohol consumption for ALBP should be interpreted cautiously.

Regarding other variables, age showed only a suggestive trend in regression sensitivity analysis, with coefficients of −0.02 points and −0.45% for absolute and relative pain change, respectively, although both are clinically negligible. The impact of age on LBP recovery remains controversial, with limited clinical relevance observed in middle-aged populations but significant effects reported in those aged ≥65 years [53,54]. Hence, our results may reflect the age restriction to individuals aged ≤65 years. This restriction likely enhanced the reliability of our findings by minimizing the confounding effects of age-related degenerative changes and comorbidities observed in older adults [23,24]. However, this approach inevitably limits the external validity of our findings to relatively younger and healthier populations. Although specific evidence for ALBP is limited, advancing age is well known to be associated with higher prevalence of LBP and greater disability [55]. Consequently, generalizability of our findings may be limited, and caution is warranted when extrapolating the results to older adults in clinical settings, as advanced age may negatively impact LBP recovery.

Furthermore, past history, such as the history of DM and PSY, often linked to poor LBP recovery, showed no consistent associations in this study, nor did HTN, which had been reported to predict better prognosis [13]. This discrepancy may be partly explained by the study design focusing on the short-term prognosis of ALBP, whereas prior research examined long-term pain outcomes over months to years, which may better capture the impact of medical history. Indeed, type 2 diabetes is associated with low-grade systemic inflammation, a factor that contributes to the development of CLBP and may necessitate prolonged periods for symptom manifestation [56,57]. Furthermore, depression and CLBP share common neurotransmitters, notably serotonin, which modulate both the descending pain inhibitory pathway and emotion regulation [58]. The clinical effects of these changes often take extended periods to emerge due to neuroplasticity [59]. Therefore, the aforementioned past history may exert a progressively greater influence on LBP recovery, with PSY in particular representing a key factor [58]. These findings underscore the necessity of long-term follow-up in future studies to fully elucidate these associations.

Hospitalization length and symptom duration demonstrated strong significance across all pain outcomes in univariate analyses and exhibited non-linear relationships in regression models. Both variables showed a rapid pain improvement trend up to 5–7 days, followed by a gradual recovery until 15–20 days. However, unlike symptom duration, hospitalization length showed a potential pain rebound beyond 20 days, suggesting important clinical implications. While short-term bed rest of 2–3 days is generally considered most effective for ALBP [60], the continued improvement observed up to 20 days in this study may reflect a synergistic effect of bed rest and active therapeutic intervention during admission. Nevertheless, the negative consequences of reduced physical activity may outweigh the benefits beyond a certain point, warranting caution in considering prolonged hospitalization. Hence, results beyond 16 days for both variables should be interpreted with caution since sparse observations in this interval led to wider confidence intervals, potentially compromising the precision of the estimates.

The present findings suggest several clinical implications. Systemic symptoms in ALBP patients, particularly dyspepsia, may serve as early indicators of slower recovery, underscoring the importance of careful assessment at the initial clinical evaluation. Identifying such patients can help clinicians to prioritize early intervention and design multidimensional management strategies aimed at preventing prolonged disability. In this context, management focusing solely on pain control may result in delayed improvement for patients presenting with dyspeptic symptoms. Therefore, combining physiotherapy or acupuncture with medication for dyspepsia may offer a viable therapeutic approach, addressing both systemic and musculoskeletal components. More broadly, incorporating symptom profiling into routine assessment may enhance prognostic accuracy and facilitate tailored treatment planning. For example, considering the combined patterns of GI symptoms, PSY, and DM alongside pain intensity could enable more precise risk stratification, providing a practical foundation for more proactive and comprehensive approaches to ALBP care.

Hence, this study has some limitations. First, the results present only correlational analyses between systemic symptoms and LBP without establishing causality. Despite outlining possible mechanisms, they remain hypothetical and require further investigation to confirm causality. Second, systemic symptoms were identified based on patients’ subjective reports in medical records rather than standardized assessment tools or objective diagnostic criteria. The reliance on subjective reports may have introduced measurement bias, potentially affecting patient selection and over- or underestimating associations with LBP. Future studies should employ validated tools, such as the Nepean Dyspepsia Index for dyspepsia or the Pittsburgh Sleep Quality Index for sleep disturbances, to enhance the reliability and specificity of systemic symptom assessment. Third, the generalizability of our findings may be limited. The present study focused solely on ALBP, with a mean total course of approximately 9 days (sum of symptom duration and hospitalization), thereby excluding CLBP populations. Despite the evidence that short-term recovery patterns can predict LBP chronicity, it remains unclear whether these associations extend to longer-term courses in CLBP patients, limiting the applicability of our findings. In addition, the exclusion of non-mechanical LBP patients, who often present with more severe or persistent pain and poorer overall health, may have led to an underestimation of symptom impact. Moreover, 93% of the included patients were diagnosed with lumbar sprain or strain, further limiting the generalizability of our findings to other mechanical LBP types, such as acute disc herniation. Finally, the size and gender distribution of the sample may have influenced the observed associations. With a sample size of 194 and no a priori calculation, the statistical power to detect small-to-moderate effects may have been limited given the number of covariates, particularly in subgroup analysis. The male-to-female ratio was also imbalanced at approximately 40:60. The smaller number of participants in one gender may have increased estimate variability and lowered the statistical power to detect gender-related differences or interactions. Although GAM and bootstrap methods were applied to enhance reliability, certain associations may have remained undetected, warranting caution in interpreting the results. Taken together, these limitations suggest that future research should include older adults, patients with CLBP and/or non-mechanical LBP, and a more balanced sex distribution. Studies should also employ standardized symptom assessments and longer follow-up periods, ideally in large-scale prospective longitudinal trials with diverse populations.

## 5. Conclusions

This retrospective cross-sectional study is the first to identify systemic symptoms, especially dyspepsia, as potential short-term prognostic factors impeding ALBP recovery. Given the influence of early recovery patterns on long-term outcomes, these findings may serve as meaningful markers for early identification of patients at risk of LBP chronicity. Furthermore, multivariable regression analysis was employed to assess the independent effects of various factors beyond systemic symptoms, acknowledging the multifactorial nature of LBP prognosis in clinical settings. This comprehensive analysis underscores the clinical relevance of a holistic approach to LBP by demonstrating the significant association between pain and dyspepsia. Despite the limitations of our study design, the obtained results suggest a novel approach for preventing chronicity and guiding proactive treatment strategies in LBP. Moreover, this study highlights the potential effectiveness of multidimensional and integrated strategies in reducing pain and enhancing patients’ quality of life in clinical practice.

## Figures and Tables

**Table 1:** Inclusion and exclusion criteria of the study.

Definition of LBP	KCD Codes S30–S39 (Injuries to the Abdomen, Lower Back, Lumbar Spine and Pelvis) or M50–M54 (Other Dorsopathies) Related to LBP
Inclusion Criteria	Age ≤ 65 years old Duration of LBP ≤ 4 weeks Hospitalization period ≥ 3 days
Exclusion Criteria	Age > 65 years old Duration of LBP > 4 weeks Hospitalization period < 3 days Non-mechanical LBP Red flags (Cauda equina, Compression fracture, etc.) Loss of conscious at onset Lack of sufficient information regarding systemic symptoms

Abbreviation: LBP, Low Back Pain; KCD, Korean Standard Classification of Diseases.

**Table 2:** Adapted inclusion criteria for systemic symptoms based on subjective complaints in traditional Korean medicine.

Systemic Symptoms	Referenced Diagnostic Criteria	Adapted Inclusion Criteria
Sleep disturbance	DSM-5 - Difficulty initiating or maintaining sleep, or early-morning awakening, occurring ≥3 nights per week for ≥3 months - Symptoms not attributable to mental disorder, substance use, or medical condition	- Frequency of DSM-5-defined sleep disturbance symptoms ≥3 nights per week during hospitalization - Excludes cases attributable to mental disorder, medical condition (ex. nocturia) or substance use (caffeine intake)
Anorexia	None	- Patient’s dietary intake decreased to ≤50% of baseline during hospitalization
Dyspepsia	Rome IV Criteria - Presence of at least one of the following: postprandial fullness, early satiation, epigastric pain, or epigastric burning - Symptom presentation for the last 3 months, with onset ≥6 months before diagnosis - No evidence of structural disease or identifiable gastrointestinal disorder	- Occurrence of at least one Rome IV-defined symptom during hospitalization - No evidence of structural disease or identifiable gastrointestinal disorder based on past history
Constipation	Rome IV Criteria - Presence of at least two of the following: bowel movements ≤ 3 times per week, straining, hard stools, sensation of incomplete evacuation, anorectal obstruction, or manual maneuvers - Symptom presentation for the last 3 months, with onset ≥6 months before diagnosis	- Occurrence of at least two Rome IV-defined symptoms during hospitalization
Nocturia	ICS - At least one episode of awakening during the night to void Article - Clinically significant, occurring at least twice per night - Regardless of voiding frequency, clinical significance could be determined by the degree of sleep disruption	- Occurrence of voiding at least twice per night during hospitalization or - Frequency of sleep disturbance due to nocturia ≥3 times per week during hospitalization
Thirst	None	- Patient-reported thirst or increased water intake during hospitalization

Abbreviation: DSM-5, Diagnostic and Statistical Manual of Mental Disorders, Fifth Edition; ICS, International Continence Society.

**Table 3:** General characteristics of included patients.

Total number of cohorts	194	
Gender (Male/Female)	79/115	
Age (Year)	47.06 ± 12.11	
Complaint duration (Day)	2.65 ± 4.18	
Hospitalization period (Day)	6.57 ± 3.84	
Diagnosis	Sprain and strain of lumbar spine	181 (93.3%)
	Sprain and strain of other and unspecified parts of lumbar spine and pelvis	8 (4.1%)
	Lumbar and other intervertebral disc disorders with radiculopathy	5 (2.6%)
	Spinal stenosis, lumbar region	2 (1.0%)
	Other spondylosis, lumbar region	1 (0.5%)
	Low back pain, lumbar region	1 (0.5%)
	Lumbago due to displacement of intervertebral disc	1 (0.5%)
	Spondylolysis, lumbar region	1 (0.5%)
NRS AD	6.09 ± 1.46	
NRS DC	4.77 ± 2.01	
ΔNRS	−1.32 ± 1.75	
ΔNRS (%)	−21.61 ± 27.93	

Abbreviation: NRS, Numeric rating scale; NRS AD, NRS at admission; NRS DC, NRS at discharge; ΔNRS, absolute pain change = NRS DC−NRS AD; ΔNRS (%), relative pain change =((NRS DC−NRS AD)/NRS AD)×100.

**Table 4:** Univariate associations with low back pain intensity.

	Variables		NRS AD	NRS DC	ΔNRS	ΔNRS (%)
(**A**) Demographic characteristics	Gender	Male (79)	6.03 ± 1.41	4.71 ± 2.02	−1.32 ± 1.82	−21.08 ± 28.87
		Female (115)	6.14 ± 1.49	4.82 ± 2.02	−1.32 ± 1.70	−21.96 ± 27.39
		*p*-value	0.801	0.693	0.819	0.759
	Age	Correlation coefficient	0.013	0.050	0.035	0.041
		*p*-value	0.858	0.491	0.630	0.634
	Smoking	Yes (28)	5.96 ± 1.53	3.93 ± 1.90	−2.04 ± 2.13	−31.99 ± 32.05
		No (166)	6.11 ± 1.45	4.92 ± 2.00	−1.20 ± 1.65	−19.86 ± 26.89
		*p*-value	0.546	0.017 *	0.051 †	0.047 *
	Alcohol	Yes (57)	6.04 ± 1.49	4.25 ± 2.17	−1.79 ± 1.81	−30.00 ± 27.78
		No (137)	6.12 ± 1.45	4.99 ± 1.91	−1.12 ± 1.69	−18.11 ± 27.34
		*p*-value	0.549	0.007 **	0.018 *	0.009 **
(**B**) Pain characteristics	NRS AD	Correlation coefficient	-	0.506	−0.145	−0.012
		*p*-value	-	0.000 ***	0.044 *	0.863
	Symptom duration	Correlation coefficient	–0.072	–0.266	−0.280	−0.302
		*p*-value	0.320	0.000 ***	0.000 ***	0.000 ***
	Hospitalization length	Correlation coefficient	0.022	–0.181	−0.215	−0.199
		*p*-value	0.761	0.011 *	0.003 **	0.005 **
	Distribution of LBP	Widespread (131)	6.24 ± 1.46	4.80 ± 1.97	−1.44 ± 1.73	−22.91 ± 26.36
		Localized (63)	5.79 ± 1.43	4.71 ± 2.11	−1.08 ± 1.76	−18.90 ± 31.00
		*p*-value	0.067 †	0.725	0.156	0.255
	ROM	Normal (81)	5.72 ± 1.39	4.48 ± 2.09	−1.23 ± 1.62	−22.85 ± 29.34
		Abnormal (113)	6.36 ± 1.45	4.98 ± 1.94	−1.38 ± 1.83	−20.71 ± 26.97
		*p*-value	0.007 **	0.089 †	0.781	0.660
(**C**) Systemic symptoms	Sleep disturbance	Yes (114)	6.20 ± 1.42	5.01 ± 2.09	−1.19 ± 1.68	−19.78 ± 26.91
		No (80)	5.94 ± 1.51	4.44 ± 1.86	−1.50 ± 1.83	−24.21 ± 29.30
		*p*-value	0.262	0.049 *	0.215	0.215
	Anorexia	Yes (47)	6.17 ± 1.36	4.96 ± 1.89	−1.21 ± 1.47	−19.95 ± 22.57
		No (147)	6.07 ± 1.49	4.71 ± 2.05	−1.35 ± 1.83	−22.13 ± 29.49
		*p*-value	0.942	0.592	0.848	0.813
	Constipation	Yes (8)	6.88 ± 1.46	6.13 ± 2.10	−0.75 ± 1.83	−9.97 ± 26.96
		No (186)	6.06 ± 1.45	4.72 ± 1.99	−1.34 ± 1.74	−22.11 ± 27.93
		*p*-value	0.133	0.076 †	0.311	0.213
	Dyspepsia	Yes (68)	6.15 ± 1.56	5.16 ± 2.06	−0.99 ± 1.52	−16.34 ± 24.81
		No (126)	6.06 ± 1.41	4.56 ± 1.97	−1.50 ± 1.84	−24.45 ± 29.18
		*p*-value	0.771	0.091	0.052 †	0.054 †
	Nocturia	Yes (65)	6.22 ± 1.39	4.88 ± 1.89	−1.34 ± 1.88	−20.59 ± 26.91
		No (129)	6.03 ± 1.49	4.72 ± 2.08	−1.31 ± 1.68	−22.12 ± 28.52
		*p*-value	0.581	0.644	0.822	0.733
	Thirst	Yes (76)	6.09 ± 1.56	4.80 ± 2.03	−1.29 ± 1.78	−21.24 ± 28.40
		No (118)	6.09 ± 1.40	4.75 ± 2.01	−1.34 ± 1.73	−21.84 ± 27.74
		*p*-value	0.680	0.887	0.744	0.820

Annotation: Data presented as mean ± standard deviation. *p*-values were calculated by Mann–Whitney U test, except for age, NRS AD, complaint duration and hospitalization period, which were analyzed by Spearman correlation analysis. († = *p* < 0.1, * = *p* < 0.05, ** *p* < 0.01, *** = *p* < 0.001). Abbreviation: NRS, Numeric rating scale; NRS AD, NRS at admission; NRS DC, NRS at discharge; ΔNRS, absolute pain change = NRS DC−NRS AD; ΔNRS (%), relative pain change =((NRS DC−NRS AD)/NRS AD)×100; LBP, Low back pain; ROM, Range of motion.

**Table 5:** The relationship between low back pain characteristics and pain intensity.

Variables	NRS DC			ΔNRS			ΔNRS (%)		
**Parametric Terms**	**Estimate**	***p*** **-Value**	**95% CI**	**Estimate**	***p*** **-Value**	**95% CI**	**Estimate**	***p*** **-Value**	**95% CI**
NRS AD	0.71	0.000 ***	(0.56, 0.90) ‡	-	-	-	-	-	-
Sleep disturbance	0.29	0.223	(−0.18, 0.77)	0.24	0.315	(−0.20, 0.76)	2.82	0.465	(−5.18, 10.27)
Dyspepsia	0.55	0.026 *	(0.05, 1.02) ‡	0.54	0.033 *	(0.06, 1.00) ‡	8.15	0.045 *	(0.27, 16.38) ‡
Constipation	0.41	0.483	(−0.95, 1.69)	0.21	0.726	(−1.29, 1.53)	7.54	0.436	(−13.35, 26.91)
Lower GI	−0.23	0.539	(−0.92, 0.43)	−0.05	0.904	(−0.75, 0.57)	−3.07	0.609	(−14.49, 7.53)
MSTL	−0.28	0.355	(−0.95, 0.35)	−0.40	0.195	(−1.07, 0.28)	−4.86	0.321	(−15.59, 5.33)
Distribution of LBP	0.03	0.908	(−0.47, 0.56)	−0.08	0.748	(−0.60, 0.41)	−0.54	0.899	(−7.91, 8.98)
Smoking	−0.60	0.10	(−1.43, 0.11)	−0.64	0.091 †	(−1.54, 0.13)	−5.22	0.384	(−19.74, 6.67)
Alcohol	−0.58	0.037 *	(−1.06, −0.03) ‡	−0.50	0.077 †	(−1.01, 0.05)	−11.57	0.011 *	(−19.33, −2.97) ‡
ROM	0.18	0.447	(−0.31, 0.64)	0.03	0.905	(−0.46, 0.49)	4.65	0.229	(−3.67, 12.44)
**Smooth terms**	**edf**	**F-value**	***p*** **-value**	**edf**	**F-value**	***p*** **-value**	**edf**	**F-value**	***p*** **-value**
Hospitalization length	3.80	3.82	0.004 **	3.96	3.56	0.005 **	3.72	2.85	0.022 **
Symptom duration	3.35	4.24	0.003 **	3.45	3.75	0.006 **	3.00	4.98	0.001 **
**Model fit summary**	*n* = 194 R^2^ (adjusted) = 0.417 Deviance explained = 46.9%			*n* = 194 R^2^ (adjusted) = 0.176 Deviance explained = 24.6%			*n* = 194 R^2^ (adjusted) = 0.168 Deviance explained = 23.6%		

Annotation: Results from multiple regression using a Generalized Additive Model (GAM). NRS AD was excluded from ΔNRS and ΔNRS (%) models to avoid mathematical coupling, as it is a component of both outcome definitions. Non-linearity was observed in Hospitalization length and Symptom duration, for which smooth terms with k = 7 were applied. Due to violations of residual normality and homoscedasticity, all models were bootstrapped. *p*-values were obtained from GAM outputs († = *p* < 0.1, * = *p*  <  0.05; ** = *p*  <  0.01; *** = *p*  <  0.001). The 95% confidence intervals were estimated via non-parametric bootstrapping and considered statistically significant if excluding zero (‡ = 95% CI excludes zero). Abbreviation: NRS, Numeric rating scale; NRS AD, NRS at admission, NRS DC, NRS at discharge; ΔNRS, absolute pain change = NRS DC − NRS AD; ΔNRS (%), relative pain change = ((NRS DC − NRS AD)/NRS AD) × 100; CI, Confidence Interval; Lower GI, Past history of Lower Gastrointestinal disease; MSTL, Past history of Musculoskeletal disease–thoracic or lumbar spinal region; LBP, Lower back pain; ROM, Range of motion.

## Data Availability

Data are not available due to ethical reasons.

## Supplementary Materials

The following supporting information can be downloaded at: https://www.mdpi.com/article/10.3390/jcm14196969/s1, Figure S1: Residuals Vs. Fitted plots for the initial multiple linear regression model; Figure S2: Partial residual plots of continuous variables in the initial multiple linear; Table S1: Association between Past Medical History and Pain Intensity; Table S2: Association between systemic symptoms and pain characteristics; Table S3: Sensitivity and Subgroup Analyses of between low back pain characteristics and pain intensity; Table S4: Results of association between binary indices included in the regression.

## Abbreviations

The following abbreviations are used in this manuscript:

LBP	Low Back Pain
ALBP	Acute Low Back Pain
CLBP	Chronic Low Back Pain
GI	Gastrointestinal
TKM	Traditional Korean Medicine
IRB	Institutional Review Board
HTN	Hypertension
DM	Diabetes Mellitus
PSY	Psychological history
ROM	Range of Motion
NRS	Numeric Rating Scale
NRS AD	Numeric Rating Scale at Admission
NRS DC	Numeric Rating Scale at Discharge
GAM	Generalized Additive Model
VIF	Variance Inflation Factors
IAP	Intra-abdominal Pressure
ACC	Anterior Cingulate Cortex
