# Supplementary material for: Association Between Systemic Symptoms and Recovery in Acute Low Back Pain: A Retrospective Cross-Sectional Study
Source: J Clin Med. 2025 Oct 1;14(19):6969. doi: 10.3390/jcm14196969 (PMC12524731; doi:10.3390/jcm14196969)
Supplement: Supplementary file 1 [file jcm-14-06969-s001.zip › Supplementary Figures.pdf]

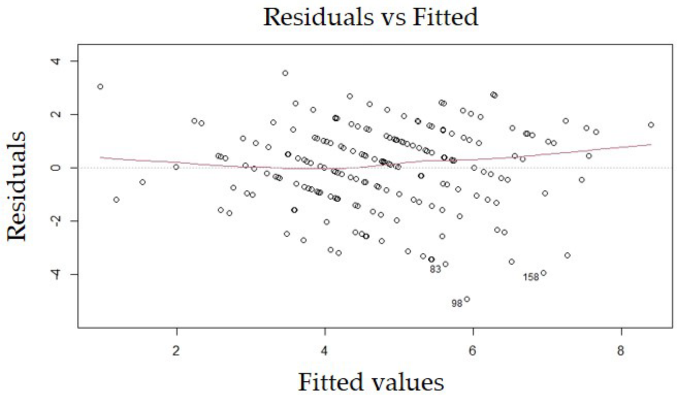

(a)

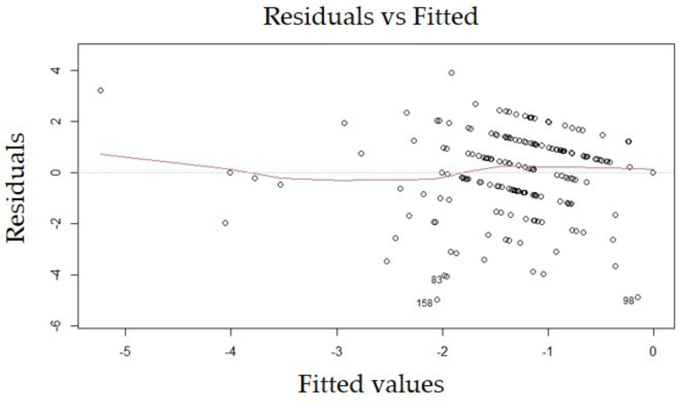

(b)

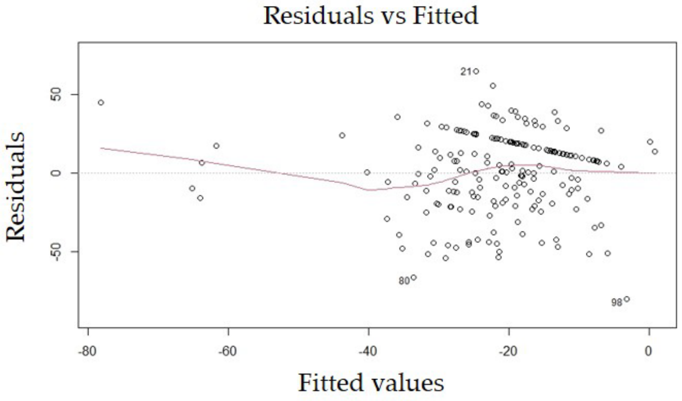

(c)

Figure S1. Residuals vs Fitted plots for the initial multiple linear regression model

The red smoothed line indicates a non-linear pattern in residuals, suggesting potential violation of the linearity assumption. Also, asymmetrical pattern of residuals suggests possible heteroscedasticity.

The presence of a striated residual pattern may reflect discrete covariates. (a) NRS DC, (b) △NRS, (c) △NRS(%)

Abbreviation: NRS, Numeric rating scale; NRS DC, NRS at discharge; △NRS, absolute pain change = NRS DC – NRS AD; △NRS(%), relative pain change =((NRS DC – NRS AD) /NRS AD) x 100

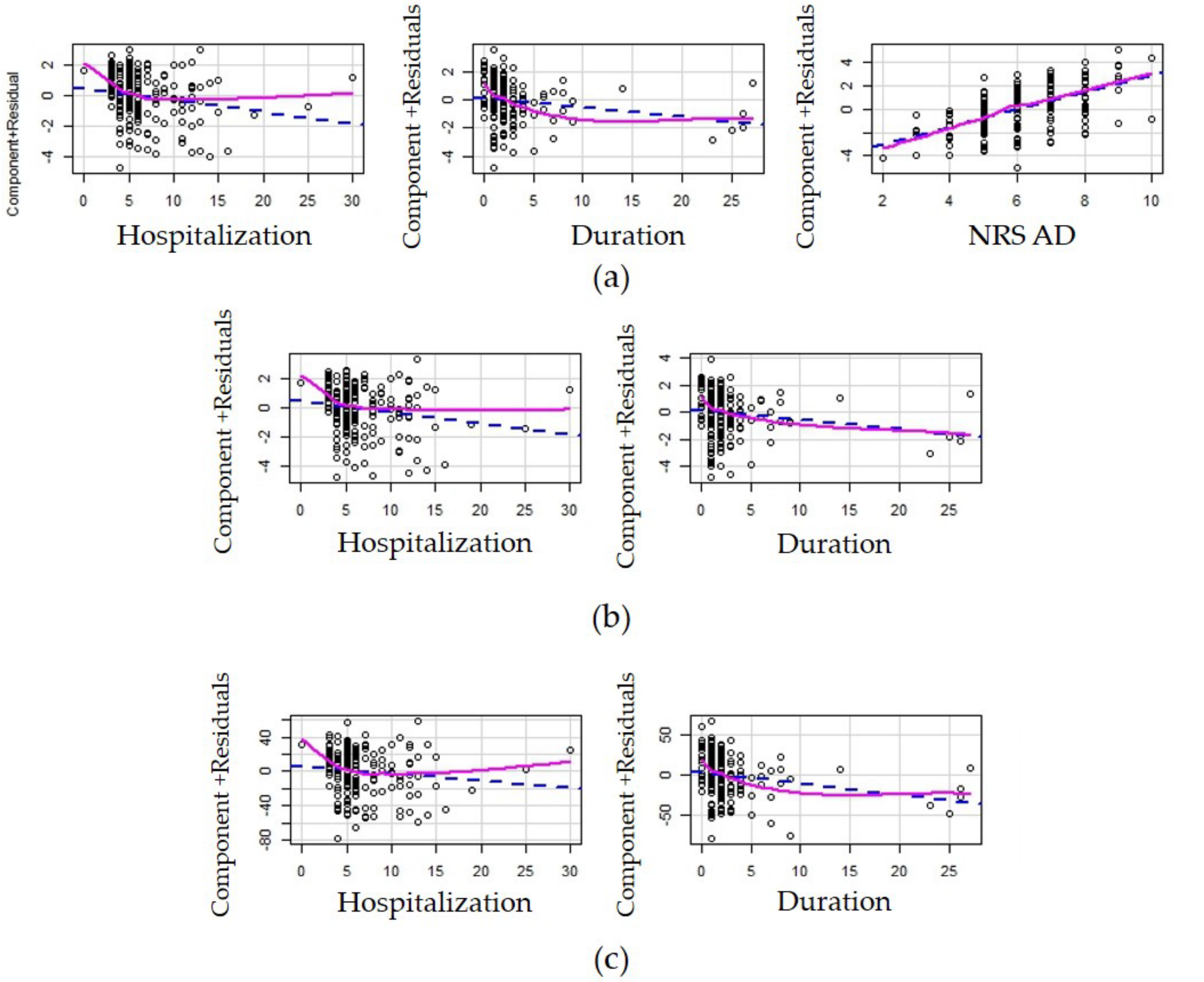

Figure S2. Partial residual plots of continuous variables in the initial multiple linear regression model. The purple lines for Hospitalization and Duration suggests potential non-linear patterns in residuals, while pain at admission shows a relatively linear relationship. (a) NRS DC, (b) △NRS, (c)△ NRS(%)

Abbreviation: NRS, Numeric rating scale; NRS AD, NRS at admission; NRS DC, NRS at discharge; △NRS, absolute pain change = NRS DC – NRS AD; △NRS(%), relative pain change =((NRS DC – NRS AD) /NRS AD) x 100
