# Supplementary material for: Association Between Systemic Symptoms and Recovery in Acute Low Back Pain: A Retrospective Cross-Sectional Study
Source: J Clin Med. 2025 Oct 1;14(19):6969. doi: 10.3390/jcm14196969 (PMC12524731; doi:10.3390/jcm14196969)
Supplement: Supplementary file 1 [file jcm-14-06969-s001.zip › Supplementary tables.pdf]

Supplementary tables

Table S1. Association between Past medical history and Pain intensity

		NRS AD	NRS DC	△NRS	△NRS (%)
Diabetes Mellitus	Yes (18)	6.11±1.45	4.78±2.02	-1.33±1.81	-21.29±28.91
	No (176)	6.09±1.46	4.77±2.02	-1.32±1.74	-21.64±27.91
	p-value	0.928	0.989	0.984	0.964
Hypertension	Yes (38)	6.08±1.65	4.89±2.15	-1.18±1.86	-19.04±28.10
	No (156)	6.10±1.41	4.74±1.98	-1.35±1.72	-22.23±27.95
	p-value	0.644	0.800	0.403	0.467
Dyslipidemia	Yes (40)	6.23±1.51	4.93±2.11	-1.30±1.84	-20.66±27.22
	No (154)	6.06±1.45	4.73±1.99	-1.32±1.73	-21.85±28.19
	p-value	0.630	0.725	0.841	0.846
Psychological history	Yes (5)	7.20±1.79	5.60±1.34	-1.60±2.30	-17.94±27.69
	No (189)	6.06±1.44	4.75±2.03	-1.31±1.74	-21.70±28.00
	p-value	0.145	0.291	0.814	0.831
Cardiovascular disease	Yes (12)	6.17±1.03	4.92±1.98	-1.25±1.71	-20.56±27.11
	No (182)	6.09±1.48	4.76±2.02	-1.32±1.75	-21.68±28.06
	p-value	0.664	0.801	0.888	0.897
Respiratory disease	Yes (7)	5.86±1.35	4.29±1.50	-1.57±1.27	-27.19±20.68
	No (187)	6.10±1.46	4.79±2.03	-1.31±1.76	-21.40±28.19
	p-value	0.653	0.519	0.449	0.414
Hepatobilary disease	Yes (11)	6.09±1.64	4.55±1.29	-1.55±2.34	-20.50±29.12
	No (183)	6.09±1.45	4.79±2.05	-1.31±1.71	-21.67±27.94
	p-value	0.634	0.704	0.923	0.937
Upper gastrointestinal disease	Yes (13)	6.46±1.45	5.54±2.22	-0.923±1.55	-14.98±25.85
	No (181)	6.07±1.46	4.72±1.99	-1.35±1.76	-22.08±28.08
	p-value	0.202	0.138	0.368	0.311
Lower gastrointestinal disease	Yes (23)	5.57±1.31	4.48±1.90	-1.09±1.35	-20.88±24.46
	No (171)	6.16±1.47	4.81±2.03	-1.35±1.79	-21.70±28.43
	p-value	0.099†	0.461	0.678	0.992
Gyneocological disease	Yes (27)	6.41±1.76	4.81±2.48	-1.59±2.04	-26.05±28.44
	No (167)	6.04±1.40	4.77±1.94	-1.28±1.70	-20.89±27.87
	p-value	0.352	0.823	0.596	0.414
Musculoskeletal disease – thoracic or lumbar spinal region	Yes (38)	6.47±1.39	4.84±2.33	-1.63±2.10	-25.15±30.97
	No (156)	6.00±1.46	4.76±1.94	-1.24±1.65	-20.74±27.18
	p-value	0.047*	0.947	0.484	0.537
Musculoskeletal disease – cervical region	Yes (9)	5.67±1.66	4.44±1.94	-1.22±1.09	-26.59±32.79
	No (185)	6.11±1.45	4.79±2.02	-1.32±1.77	-21.36±27.75
	p-value	0.643	0.861	0.768	0.693
Musculoskeletal disease – non-spinal region	Yes (24)	6.50±1.79	4.92±2.30	-1.58±2.12	-22.19±31.27
	No (170)	6.04±1.40	4.75±1.98	-1.28±1.69	-21.52±27.53
	p-value	0.171	0.788	0.767	0.978

Annotation: Data presented as mean ± standard deviation. *p*-values were calculated by Mann-whitney U test (†=p<0.1, *=p<0.05).

Abbreviation : NRS, Numeric rating scale; NRS AD, NRS at admission, NRS DC, NRS at discharge; △NRS, absolute pain change = NRS DC – NRS AD; △NRS(%), relative pain change =((NRS DC – NRS AD) /NRS AD) x 100

Table S2. Association between systemic symptoms and pain characteristics

		Distribution of LBP			ROM		
		Widespread (%)	Localized (%)	*p*-value	Normal (%)	Abnormal (%)	*p*-value
Sleep disorder	Yes	82 (71.9)	32 (28.1)	0.118	45 (39.5)	69 (60.5)	0.442
	No	49 (61.3)	31 (38.8)		36 (45.0)	44 (55.0)	
Anorexia	Yes	36 (76.6)	11 (23.4)	0.127	16 (34.0)	31 (66.0)	0.218
	No	95 (64.6)	52 (35.4)		65 (44.2)	82 (55.8)	
Constipation	Yes	8 (100)	0 (0)	0.055†	3 (37.5)	5 (62.5)	1.000
	No	123 (66.1)	63 (33.9)		78 (41.9)	108 (58.1)	
Dyspepsia	Yes	52 (76.5)	16 (23.5)	0.051†	29 (42.6)	39 (57.4)	0.853
	No	79 (62.7)	47 (37.3)		52 (41.3)	74 (58.7)	
Nocturia	Yes	49 (75.4)	16 (24.6)	0.097	22 (33.8)	43 (66.2)	0.113
	No	82 (63.6)	47 (36.4)		59 (45.7)	70 (54.3)	
Thirst	Yes	51 (67.1)	25 (32.9)	0.920	32 (42.1)	44 (57.9)	0.936
	No	80 (67.8)	38 (32.2)		49 (41.5)	69 (58.5)	

Annotation: Data presented as N (%). *p*-values were calculated by the chi-squared test, except for those between constipation and pain characteristics, which were calculated using Fisher's exact test. (†=$p<0.1$).
Abbreviation: LBP, Low back pain; ROM, Range of motion

Table S3. Sensitivity and subgroup analyses of between low back pain characteristics and pain intensity

Variables		NRS DC				△NRS				△NRS(%)			
Parametric terms		Base	All indices	Reported indices	Lumbar sprain	Base	All indices	Reported indices	Lumbar sprain	Base	All indices	Reported indices	Lumbar sprain
NRS AD	Estimate	0.71	0.71	0.71	0.71	N/A	N/A	N/A	N/A	N/A	N/A	N/A	N/A
	p-value	0.000***	0.000***	0.000***	0.000***								
Dyspepsia	Estimate	0.55	0.66	0.69	0.49	0.54	0.67	0.69	0.48	8.15	10.21	10.43	7.23
	p-value	0.026*	0.014*	0.007**	0.056†	0.033*	0.016*	0.009*	0.068†	0.045*	0.022*	0.014*	0.088†
Alcohol	Estimate	-0.58	-0.66	-0.70	-0.49	-0.50	-0.57	-0.63	-0.41	-11.57	-13.13	-13.64	-10.70
	p-value	0.037*	0.025*	0.014*	0.090†	0.077†	0.067†	0.031*	0.172	0.011**	0.007**	0.004**	0.026*
Smoking	Estimate	-0.60	-0.72	-0.75	-0.56	-0.64	-0.75	-0.77	-0.62	-5.22	-8.40	-8.45	-4.38
	p-value	0.100	0.067†	0.047*	0.139	0.091†	0.063†	0.052†	0.115	0.384	0.196	0.177	0.486
Age	Estimate	N/A	-0.02	-0.02	N/A	N/A	-0.02	-0.02	N/A	N/A	-0.45	-0.31	N/A
	p-value		0.096†	0.097†			0.175	0.121			0.050†	0.09†	
ROM	Estimate	0.18	0.30	0.30	0.21	0.03	0.11	0.14	0.05	4.65	6.84	6.62	5.12
	p-value	0.447	0.251	0.224	0.387	0.905	0.679	0.563	0.830	0.229	0.105	0.100†	0.208
Smooth terms		Base	All indices	Reported indices	Lumbar sprain	Base	All indices	Reported indices	Lumbar sprain	Base	All indices	Reported indices	Lumbar sprain
Hospitalization length	edf	3.801	3.565	3.885	2.445	3.956	3.648	3.986	2.487	3.724	3.568	3.825	2.683
	F-value	3.821	4.271	3.766	3.052	3.562	3.790	3.445	3.102	2.845	3.185	2.911	2.446
	p-value	0.004**	0.002**	0.004**	0.010*	0.005**	0.005**	0.007*	0.020*	0.022**	0.014**	0.019**	0.049*
Symptom duration	edf	3.349	3.386	3.489	3.018	3.446	3.493	3.547	3.116	2.997	3.120	3.219	2.691
	F-value	4.241	5.114	5.140	3.655	3.746	4.562	4.583	3.764	4.980	5.588	5.803	4.337
	p-value	0.003**	0.001**	0.001**	0.013*	0.006**	0.002**	0.002**	0.033*	0.001**	0.000***	0.000***	0.004**
Model fit summary	N	194	194	194	181	194	194	194	181	194	194	194	181
	R^2	0.417	0.399	0.422	0.400	0.176	0.151	0.181	0.108	0.168	0.143	0.179	0.116
	Deviance explained	46.9%	50.1%	48.9%	45.2%	24.6%	29.2%	27.3%	18.0%	23.6%	28.4%	26.9%	18.8%

Annotation:

Results from sensitivity and subgroup analysis using a Generalized Additive Model (GAM). NRS AD was excluded from ΔNRS and ΔNRS(%) models to avoid mathematical coupling, as it is a component of both outcome definitions. Non-linearity was observed in Hospitalization and Duration, for which smooth terms with k=7 were applied. Bootstrapping was omitted, as the analysis focused on consistency in coefficient direction and p-values, despite residual assumption violations. *p*-values were obtained from GAM outputs (†=p<0.1, *=p < 0.05; **=p < 0.01; ***=p < 0.001).

Abbreviation: NRS, Numeric rating scale; NRS AD, NRS at admission, NRS DC, NRS at discharge; △NRS, absolute pain change = NRS DC – NRS AD; △NRS(%), relative pain change =((NRS DC – NRS AD) /NRS AD) x 100; N/A, Not available; ROM, Range of motion.

Table S4. Results of association between binary indices included in the regression

	Sleep disorder	Dyspepsia	Constipation	Lower GI	MSTL	Widespread	Smoking	Alcohol	ROM
Sleep disorder	-	0.879	0.144	0.653	0.204	0.164	1.000	0.523	0.182
Dyspepsia		-	0.022*	0.356	0.924	0.043*	0.450	0.342	0.986
Constipation			-	0.599▲	0.610	0.044*	0.237	0.788	0.055
Lower GI				-	0.049*	0.795	0.659	0.533	0.197
MSTL					-	0.035*	0.453	0.250	0.053
Widespread						-	0.605	0.114	0.418
Smoking							-	0.000**	0.786
Alcohol								-	0.159
ROM									-

Annotation:
p-values were calculated by chi-squared test, except for the association between constipation and Lower GI (*=p<0.05, **=p<0.001)
▲Association between constipation and Lower GI was evaluated using fisher's exact test.

Abbreviation: Lower GI, Past history of lower gastrointestinal disease; MSTL, Past history of Musculoskeletal disease-thoracic or lumbar spinal region; ROM, Range of motion.
